# Effects of Tai Chi combined with repetitive transcranial magnetic stimulation on sleep and emotional outcomes in older adults: A systematic review and meta-analysis of randomized controlled trials

**DOI:** 10.1097/MD.0000000000051253

**Published:** 2026-11-06

**Authors:** Mengxia Xu, Yu Wang, Zhoujian Ding, Jianxia Zhuang, Shengen Zheng

**Affiliations:** a Department of Geriatric Psychiatry, Taizhou Second People’s Hospital, Taizhou, Zhejiang, China; b Stroke Center, Yuhuan People’s Hospital, Yuhuan, Zhejiang, China; c Department of Internal Medicine, Shamen Town Health Clinic, Taizhou, Zhejiang, China.

**Keywords:** aged, anxiety, depression, meta-analysis, sleep wake disorders, Tai Ji, transcranial magnetic stimulation

## Abstract

**Background:**

Sleep problems are common in older adults and are frequently accompanied by emotional symptoms, including depression, anxiety, and stress. Tai Chi and repetitive transcranial magnetic stimulation (rTMS) have both been applied as non-pharmacological interventions for sleep and mood regulation. However, evidence regarding the effectiveness of their combined use remains limited.

**Methods:**

A systematic literature search was conducted in PubMed, Web of Science, Embase, the Cochrane Library, CINAHL, and Scopus from inception to December 16, 2025, to identify randomized controlled trials. The search strategy combined terms related to *Tai Chi, repetitive transcranial magnetic stimulation, and randomized controlled trials*. Reference lists of relevant articles were also screened to identify additional eligible studies. Pooled mean differences with 95% confidence intervals were generated using RevMan 5.4, with risk of bias and certainty assessed through established tools including GRADE.

**Results:**

Three trials encompassing 211 older adults qualified for analysis. Combined Tai Chi and rTMS was associated with a significant reduction in sleep severity compared with control interventions (SMD = –0.51; 95% CI: –0.90 to –0.12; *P* = .01; *I*^2^ = 43%). Evidence for other sleep-related outcomes and for depression, anxiety, and stress was inconclusive.

**Conclusion:**

The available evidence suggests that the combination of Tai Chi and rTMS may improve overall sleep severity; however, this finding should be interpreted cautiously given the small number of trials and sensitivity to individual studies. Evidence for specific sleep parameters and emotional outcomes remains inconclusive. Further well-designed randomized trials are needed to clarify the potential role of this combined intervention in sleep and mood regulation.

## 1. Introduction

Sleep disturbances are highly prevalent in middle-aged and older adults and represent an important public health concern because of their associations with impaired daytime functioning, reduced quality of life, cognitive decline, and adverse physical and psychological outcomes. Sleep problems in later life also frequently coexist with depression, anxiety, and stress, and these conditions may interact bidirectionally, further increasing the clinical burden. Given concerns regarding adverse effects, dependence, and limited long-term suitability of sedative-hypnotic medications in older populations, increasing attention has been directed toward non-pharmacological strategies for improving sleep. Recent comparative evidence indicates that a broad range of non-pharmacological interventions can improve sleep quality in adults aged ≥ 50 years, although the magnitude of benefit varies across intervention modalities.^[1]^

Tai Chi is a mind–body exercise characterized by slow and coordinated movements, postural control, regulated breathing, and focused attention. These components may influence sleep through several interconnected mechanisms, including reductions in physiological arousal, regulation of autonomic activity, improvement in emotional regulation, and promotion of regular physical activity. Importantly, Tai Chi is low impact, requires minimal equipment, and can generally be adapted to the functional capacity of older adults, giving it practical advantages over more physically demanding exercise interventions. Recent quantitative evidence supports its potential role in sleep management. A 2024 systematic review and meta-analysis found significantly better sleep quality following Tai Chi than control interventions.^[2]^

Repetitive transcranial magnetic stimulation (rTMS) represents a mechanistically distinct non-pharmacological approach. rTMS is a noninvasive neuromodulation technique that uses repeated magnetic pulses to alter cortical excitability and functional connectivity in targeted neural networks. Unlike behavioral or exercise-based interventions, rTMS directly modulates neural activity and may influence brain networks involved in sleep–wake regulation and emotional processing. Recent evidence has continued to evaluate noninvasive brain stimulation for insomnia; a 2025 systematic review examining rTMS and other noninvasive brain stimulation modalities reported potential improvements in sleep outcomes while also emphasizing heterogeneity in stimulation protocols and the need for stronger evidence.^[3]^ Thus, rTMS may provide a complementary therapeutic approach, particularly when sleep disturbance coexists with neuropsychiatric symptoms or is insufficiently responsive to behavioral strategies alone.

Recent comparative evidence provides an important context for considering these 2 interventions. Wang et al conducted a systematic review and network meta-analysis of non-pharmacological interventions for sleep quality in adults aged ≥ 50 years, demonstrating that multiple approaches can improve sleep while their relative effects vary across intervention types. However, existing comparative evidence has largely evaluated individual intervention modalities rather than combinations of mind–body exercise and neuromodulation. Tai Chi and rTMS may have complementary rather than redundant therapeutic characteristics: Tai Chi engages physical, behavioral, attentional, and autonomic regulatory pathways, whereas rTMS provides targeted modulation of cortical and network-level activity. Combining these approaches could therefore address sleep disturbance through partially distinct mechanisms. Nevertheless, the superiority of combined Tai Chi and rTMS over other non-pharmacological approaches cannot currently be established because direct comparative evidence remains limited.

Several randomized controlled trials have recently evaluated combined Tai Chi and rTMS for sleep disturbances in older adults. However, individual trials have generally involved relatively small samples and have used heterogeneous measures of sleep and emotional outcomes. Consequently, the magnitude, consistency, and certainty of the effects of this combined intervention remain unclear. Therefore, the present systematic review and meta-analysis synthesized randomized controlled trial evidence to evaluate the effects of combined Tai Chi and rTMS on sleep severity, objective sleep parameters, and emotional outcomes in older adults.

## 2. Methods

### 2.1. Eligibility criteria

This systematic review was reported in accordance with the Preferred Reporting Items for Systematic Reviews and Meta-Analyses guidelines and was prospectively registered with International Prospective Register of Systematic Reviews (CRD420251267530). Eligibility was determined using the Population, Intervention, Comparator, Outcomes, and Study design framework. Randomized controlled trials were considered eligible when they enrolled older adults with sleep disturbance or insomnia and evaluated Tai Chi in combination with repetitive transcranial magnetic stimulation (rTMS). Older adults were defined as participants aged ≥ 60 years or study populations with a reported mean age of ≥60 years. Eligible comparators included usual care, sham stimulation, exercise, and other control interventions. Studies were required to report at least one sleep-related or emotional outcome. Non-randomized studies, conference abstracts, reviews, case reports, and doctoral dissertations were excluded. Studies were also excluded when the information required for quantitative synthesis could not be obtained.

### 2.2. Search strategy

Literature was systematically searched in 6 databases (EMBASE, PubMed, Cochrane Library, Web of Science, CINAHL, and Scopus). The data were updated up to December 16, 2025. The following search strategy was utilized in the search in PubMed: (((((aging [MeSH Terms]) OR (older [Title/Abstract])) OR (elder [Title/Abstract])) AND (randomized [Title/Abstract])) AND (tai chi [Title/Abstract])) AND (repetitive transcranial magnetic stimulation [Title/Abstract]). A manual search was also conducted on each of the reference lists of the included studies. The full search strategies for all databases are provided in Supplementary Appendix 1, Supplemental Digital Content 1.

### 2.3. Study selection

Two reviewers independently screened retrieved records by title and abstract, followed by full-text assessment of potentially eligible articles. Differences in judgment were settled through discussion; a third reviewer was consulted when consensus could not be reached. The analytical procedures were informed by previously published meta-analytic approaches using standardized effect estimates, heterogeneity evaluation, and sensitivity analysis.^[4]^ For studies with more than 2 treatment arms, only groups relevant to the prespecified comparison were entered into a given quantitative synthesis. No participant contributed more than once to the same pooled analysis, thereby preventing double-counting.

### 2.4. Data extraction

Two reviewers independently collected study information using a predefined extraction form, with disagreements resolved by consensus or consultation with a third reviewer. Extracted study characteristics included author, publication year, country, intervention duration, treatment and comparator conditions, sample size, sex distribution, and mean participant age.

Sleep severity was assessed using validated measures such as the Pittsburgh Sleep Quality Index (PSQI) and Insomnia Severity Index. Actigraphy-derived outcomes included sleep efficiency, sleep latency, total sleep time, and wake after sleep onset (WASO). The number of awakenings was not quantitatively synthesized because definitions and reporting methods differed across studies. Depression, anxiety, and stress outcomes were extracted from validated instruments, including the Hamilton Rating Scale for Depression and the 21-item Depression Anxiety Stress Scales.

For continuous variables, means and standard deviations were collected. Where sufficient information was available, within-group change from baseline to post-intervention was derived, and the corresponding mean change and standard deviation were entered into the analysis. Standardized mean differences were used when studies assessed the same construct with different instruments; mean differences (MDs) were used when measurements were reported on a common scale. When studies reported several follow-up assessments, the assessment corresponding to completion of the intervention was selected.

### 2.5. Study risk of bias assessment

Two reviewers independently evaluated methodological quality using the Cochrane risk-of-bias framework in Review Manager (RevMan) 5.4. The assessment considered random sequence generation, allocation concealment, blinding, incomplete outcome data, selective reporting, and other potential sources of bias. Each domain was judged as low, high, or unclear risk.

### 2.6. Data synthesi

Quantitative analyses were performed with RevMan 5.4 using random-effects models and inverse-variance weighting. Effect estimates are reported with 95% confidence intervals (CIs). Standardized mean differences were calculated when outcome instruments differed across studies, whereas MDs were used for outcomes measured on the same scale.

Between-study heterogeneity was quantified using the *I*^2^ statistic and interpreted as low (<25%), moderate (26–50%), or high (>50%). Statistical significance was defined using a two-sided *P* value ≤ .05.

The primary analyses preferentially used change-from-baseline values. Mean change was calculated as the post-intervention value minus the baseline value. When an standard deviation for the change was not directly reported, it was derived from the baseline and post-intervention standard deviations using the prespecified formula, assuming a baseline–follow-up correlation of *R* = 0.5. When change data could not be derived reliably, post-intervention values were analyzed instead. Differences related to assessment timing were explored through subgroup analyses according to measurement time point.

### 2.7. Assessment of publication bias

Formal assessment of publication bias was not undertaken because the review included fewer than ten studies, making funnel-plot asymmetry difficult to evaluate reliably. Nevertheless, the limited number of available trials means that publication bias cannot be excluded.^[5]^

### 2.8. Ethics statement

Because this review synthesized data already available in published studies and involved neither direct participant recruitment nor collection of identifiable individual-level information, institutional review board approval and informed consent were not required.

## 3. Results

### 3.1. Studies selection

The database search yielded 24 records. Following removal of duplicates, 8 unique records underwent title and abstract screening, of which 4 were excluded. The remaining 4 articles were reviewed in full text. One article was subsequently excluded because it was a doctoral dissertation. Consequently, 3 randomized controlled trials met the eligibility criteria and were included in the quantitative synthesis (Fig. 1).

**Figure 1.**
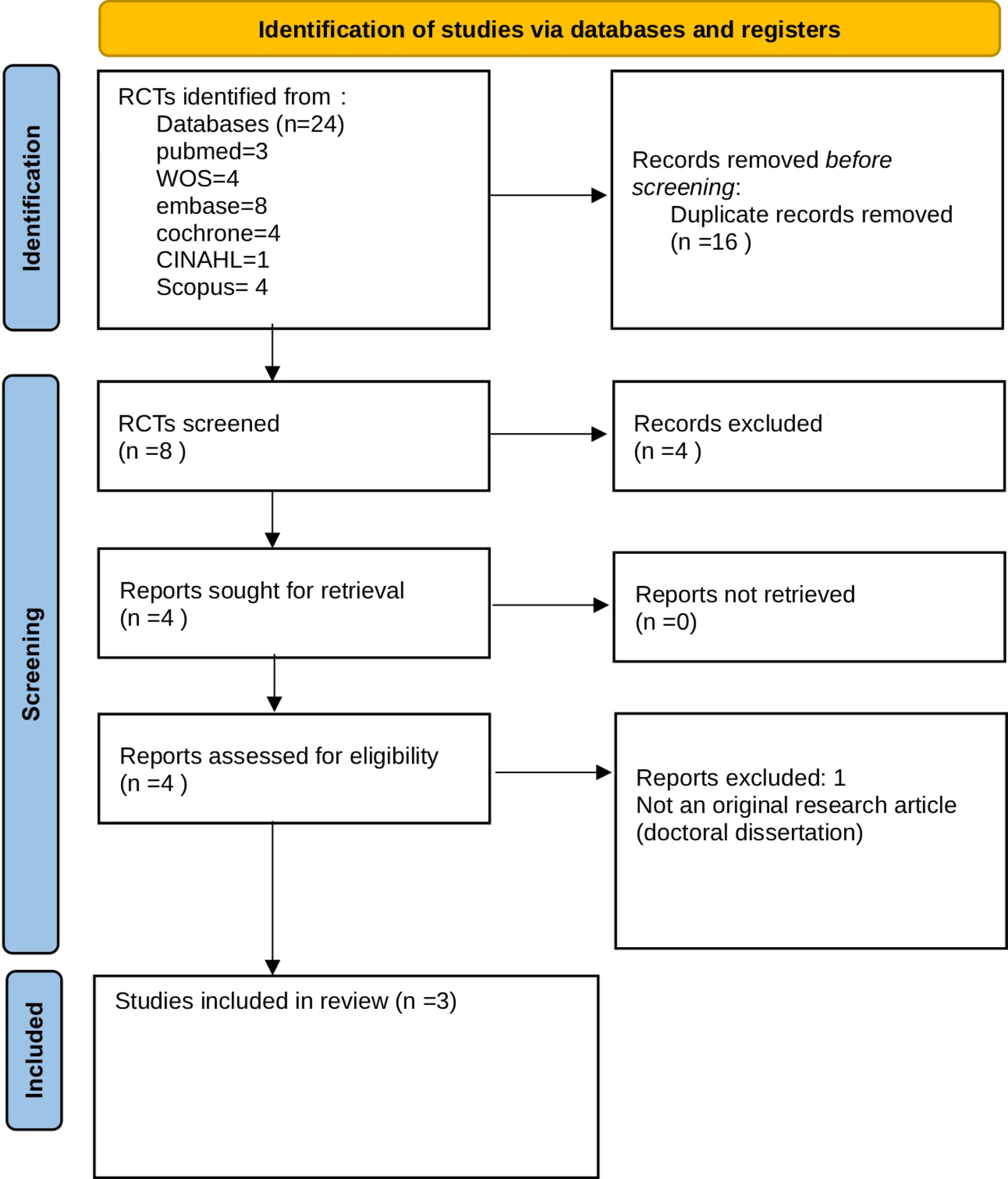
The PRISMA flow diagram. PRISMA = Preferred Reporting Items for Systematic Reviews and Meta-Analyses.

### 3.2. Characteristics of included studies

A total of 3 randomized controlled trials^[6–8]^ were included in this meta-analysis, all of which were conducted in China between 2024 and 2025. The total sample size across the included studies was 211 participants, with individual trial sample sizes ranging from 25^[7]^ to 110^[8]^ participants. All studies applied an intervention duration of 3 months, and no variation in treatment length was observed across trials.

The study populations consisted primarily of older adults experiencing sleep disturbances, with mean baseline ages of approximately 66 to 69 years. Participants in the intervention arms received Tai Chi combined with rTMS, whereas the comparator conditions varied among the included trials. Sleep severity was measured using either the PSQI or the Insomnia Severity Index.

Actigraphy was used to assess objective sleep parameters, including sleep efficiency, sleep latency, total sleep time, WASO, and number of awakenings. Emotional outcomes included measures of depression, anxiety, and stress, assessed using validated instruments such as the DASS.

Computer-based procedures were used for randomization in all 3 trials, although the reporting of methodological details differed across studies. No serious intervention-related adverse events were reported. Study characteristics and outcome data are provided in Tables 1 and 2.

**Table 1:** The characteristics of Tai Chi combined with rTMS in older adults with sleep disorders.

Study ID	Year	Country	Duration	Group	n	Sex (male)	Age	Sleep severity	Actigraphy-assessed	DASS	Randomization method
Liu	2025	China	3 mo	Tai Chi + rTMS	55	18	67.8 ± 4.5	PSQI	Sleep efficiency; Sleep latency; Total sleep time; WASO; Number of awakenings	Depression score; Anxiety score	Computer-based randomization
				Tai Chi + sham rTMS	55	24	68.0 ± 4.6				
He	2024	China	3 mo	Tai Chi + rTMS	12	9	68.17 ± 4.47	ISI	Sleep efficiency; Total sleep time; WASO	Depression score; Anxiety score; stress score	Computer-generated random number
				Tai Chi alone	13	13	69.62 ± 4.46				
Tsang	2024	China	3 mo	Tai Chi + rTMS	38	12	66.63 ± 5.32	ISI	Sleep efficiency; Sleep latency; Total sleep time; WASO; Number of awakenings	Depression score; Anxiety score; stress score	Computer-based randomization
				Tai Chi + sham rTMS	38	8	68.32 ± 4.73				

DASS = depression anxiety stress scales, ISI = Insomnia Severity Index, PSQI = Pittsburgh Sleep Quality Index, rTMS = repetitive transcranial magnetic stimulation, SD = standard deviation, TC = Tai Chi, WASO = wake after sleep onset.

**Table 2:** Changes in sleep and emotional outcomes following Tai Chi combined with rTMS.

Study	n	Group	Sleep severity	Sleep efficiency (%)	Sleep latency (min)	Total sleep time (min)	WASO (min)	Depression score	Anxiety score	Stress score
Liu, 2025	55	Tai Chi ± rTMS	−6.1 ± 4.33*	1.0 ± 7.47	−1.8 ± 37.98	−3.6 ± 47.49	5.6 ± 60.36	−3.5 ± 3.53‡ (Hamilton depression rating scale)	−4.2 ± 4.39‡ (Hamilton anxiety rating scale)	–
	55	Tai Chi + sham rTMS	−3.0 ± 4.50*	1.0 ± 7.47	0.9 ± 42.77	−13.7 ± 58.64	5.6 ± 56.41	−1.7 ± 3.40‡ (Hamilton depression rating scale)	−2.0 ± 4.41‡ (Hamilton anxiety rating scale)	–
He, 2024	12	Tai Chi ± rTMS	−9.53 ± 6.37†	2.33 ± 4.65	–	37.64 ± 103.08	−7.84 ± 18.81	−7.15 ± 7.64§ (DASS-21)	−4.78 ± 10.53§ (DASS-21)	−11.03 ± 14.86^b^ (DASS-21)
	13	Tai Chi alone	−4.07 ± 7.12†	−1.63 ± 6.0	–	−52.48 ± 108.00	1.54 ± 17.42	−1.56 ± 12.83§ (DASS-21)	0.87 ± 10.00§ (DASS-21)	0.55 ± 12.72^b^ (DASS-21)
Tsang, 2024	38	Tai Chi ± rTMS	−4.84 ± 7.51†	−0.01 ± 9.9	−4.03 ± 14.37	11.67 ± 73.22	−7.86 ± 55.69	−0.74 ± 8.80§ (DASS-21)	−0.15 ± 7.87§ (DASS-21)	−1.49 ± 10.78^b^ (DASS-21)
	38	Tai Chi + sham rTMS	−3.58 ± 7.21†	0.44 ± 11.05	−0.86 ± 13.96	10.46 ± 80.69	−1.59 ± 65.70	−2.14 ± 9.90§ (DASS-21)	−1.76 ± 9.35§ (DASS-21)	−2.76 ± 10.01^b^ (DASS-21)

Data are presented as mean change ± SD.

* PSQI = Pittsburgh Sleep Quality Index.

† ISI = Insomnia Severity Index.

‡ HAMD = Hamilton depression rating scale or HAMA, Hamilton anxiety rating scale, as applicable.

§ DASS-21 = 21-item Depression Anxiety Stress Scales.

rTMS = repetitive transcranial magnetic stimulation, SD = standard deviation, WASO = wake after sleep onset.

### 3.3. Risk of bias assessment

Figures 2 and 3 summarize the risk-of-bias assessment. Overall, methodological concerns across the included trials were generally low to moderate. Random allocation was generated using computer-based methods in all studies. Allocation concealment and assessor masking were adequately described in the larger sham-controlled trials but were less clearly documented in the pilot study.

**Figure 2.**
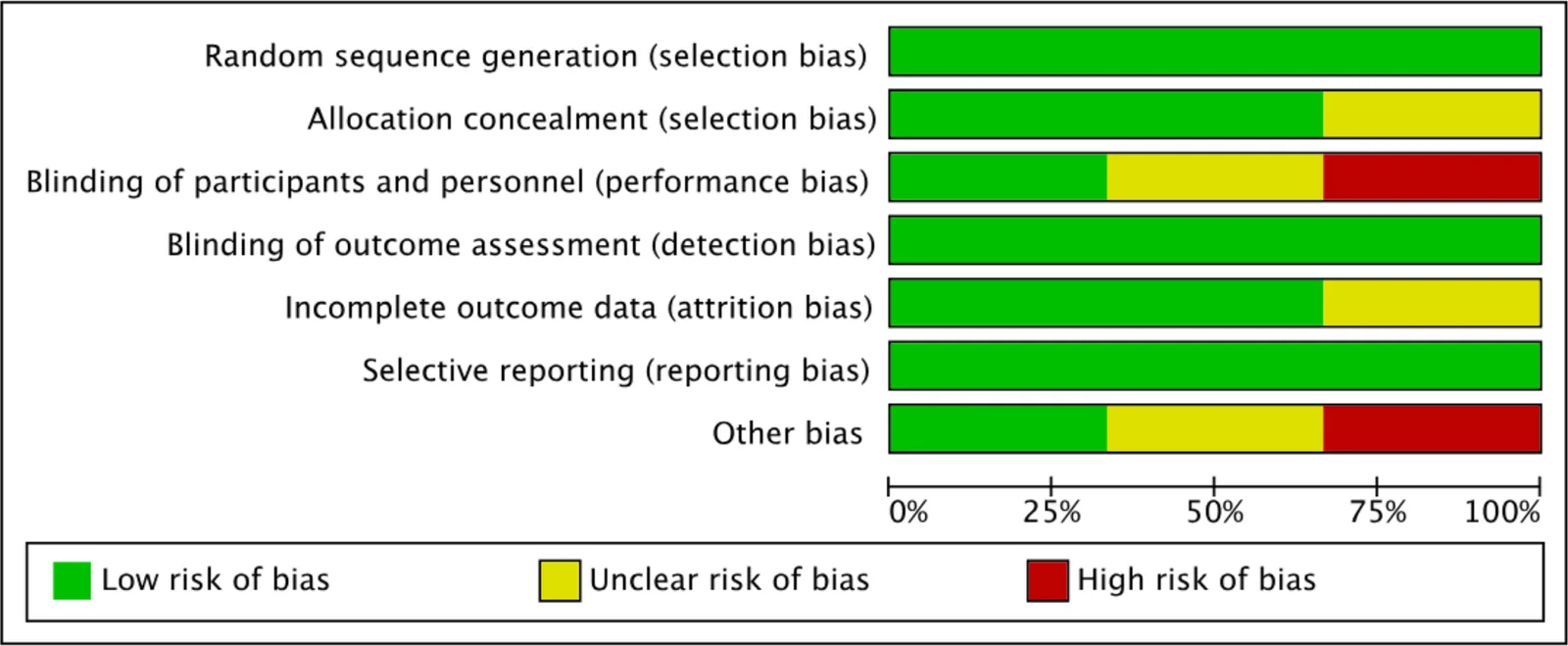
Graph for the risk of bias.

**Figure 3.**
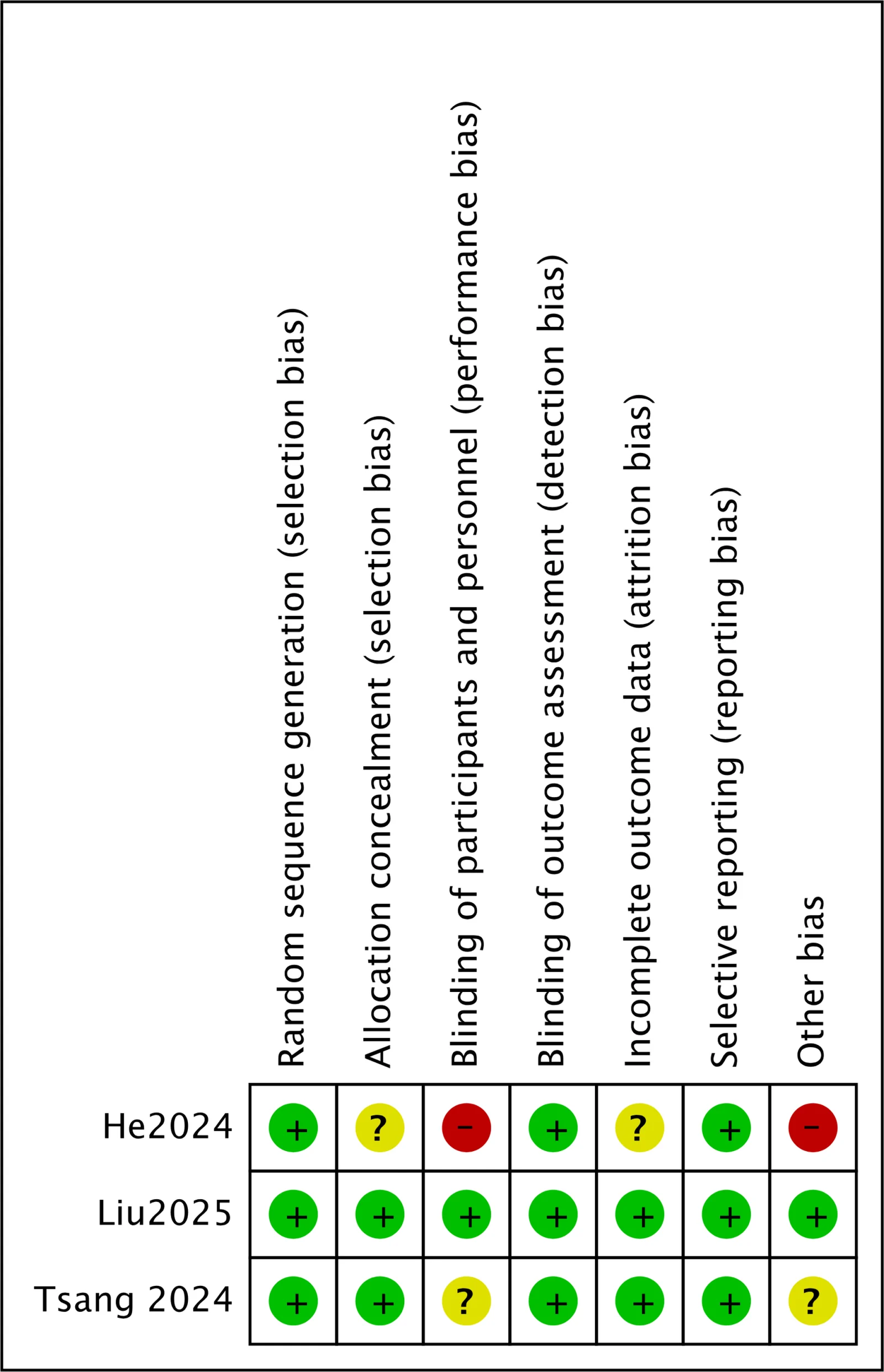
Summary of the risk of bias.

Because Tai Chi represents a behavioral intervention, complete masking of participants and intervention providers was not possible in all comparisons, introducing a potential source of performance bias. This issue was considered separately from detection bias arising from outcome assessment. In particular, uncertainty regarding assessor blinding in the pilot trial may be relevant to subjective outcomes.

Loss to follow-up was limited and generally comparable between study groups, and intention-to-treat approaches were commonly reported. The trials were prospectively registered and reported their prespecified outcomes, with little indication of selective outcome reporting. Nevertheless, the methodological limitations identified above should be considered when interpreting the findings, particularly those based on subjective sleep measures.

### 3.4. Primary outcome

Data on sleep severity were available from all 3 trials, representing 211 participants. Meta-analysis indicated lower sleep-severity scores following combined Tai Chi and rTMS than following the comparator interventions (standardized mean difference [SMD] = −0.51; 95% CI: −0.90 to−0.12; *P* = .01). Between-study heterogeneity was moderate (*I*^2^ = 43%) (Fig. 4A).

**Figure 4.**
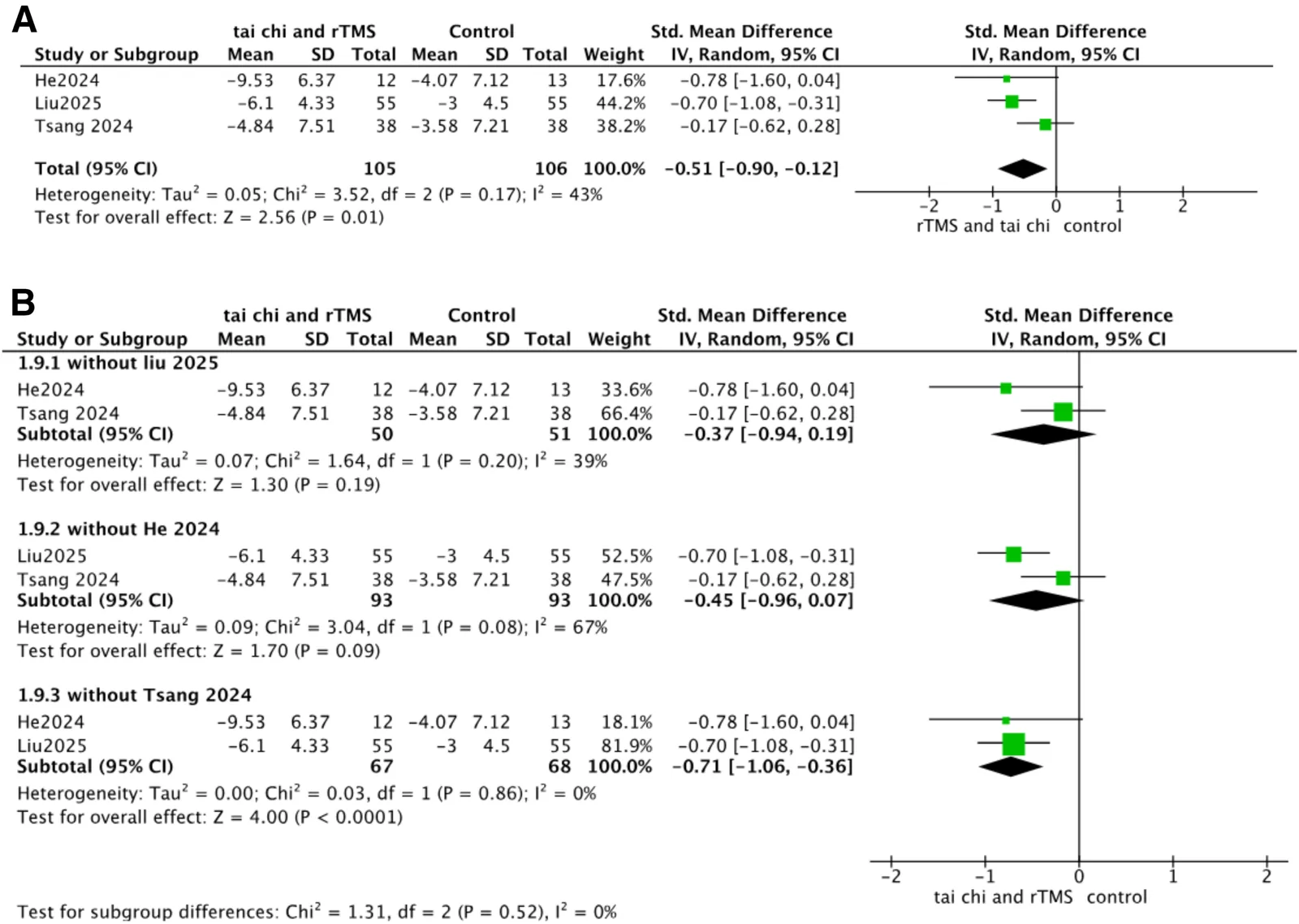
Pooled effects of Tai Chi combined with repetitive transcranial magnetic stimulation (rTMS) on sleep severity in older adults. (A) Forest plot. (B) Sensitivity analysis.

Leave-one-out analyses indicated that the pooled estimate was influenced by individual trials. Removing Liu et al (2025) reduced the effect to SMD = −0.37 (95% CI: −0.94–0.19), whereas omission of He et al (2024) resulted in an SMD of −0.45 (95% CI: −0.96–0.07); neither estimate remained statistically significant. Conversely, after excluding Tsang et al (2024), the estimated effect became larger (SMD = −0.71; 95% CI: −1.06 to −0.36), with heterogeneity decreasing to 0%. Thus, although the primary analysis favored the combined intervention, its statistical significance was sensitive to the inclusion of individual studies and should be interpreted cautiously (Fig. 4B).

### 3.5. Secondary sleep-related outcomes

#### 3.5.1. Sleep efficiency

Sleep efficiency data were available for all 211 participants across the 3 trials. The pooled mean difference between the combined intervention and comparator groups was −0.89 percentage points (95% CI: −2.98–1.19; *P* = .40), with *I*^2^ = 28%. The confidence interval crossed the null, leaving the evidence for an effect on sleep efficiency inconclusive (Fig. 5A).

**Figure 5.**
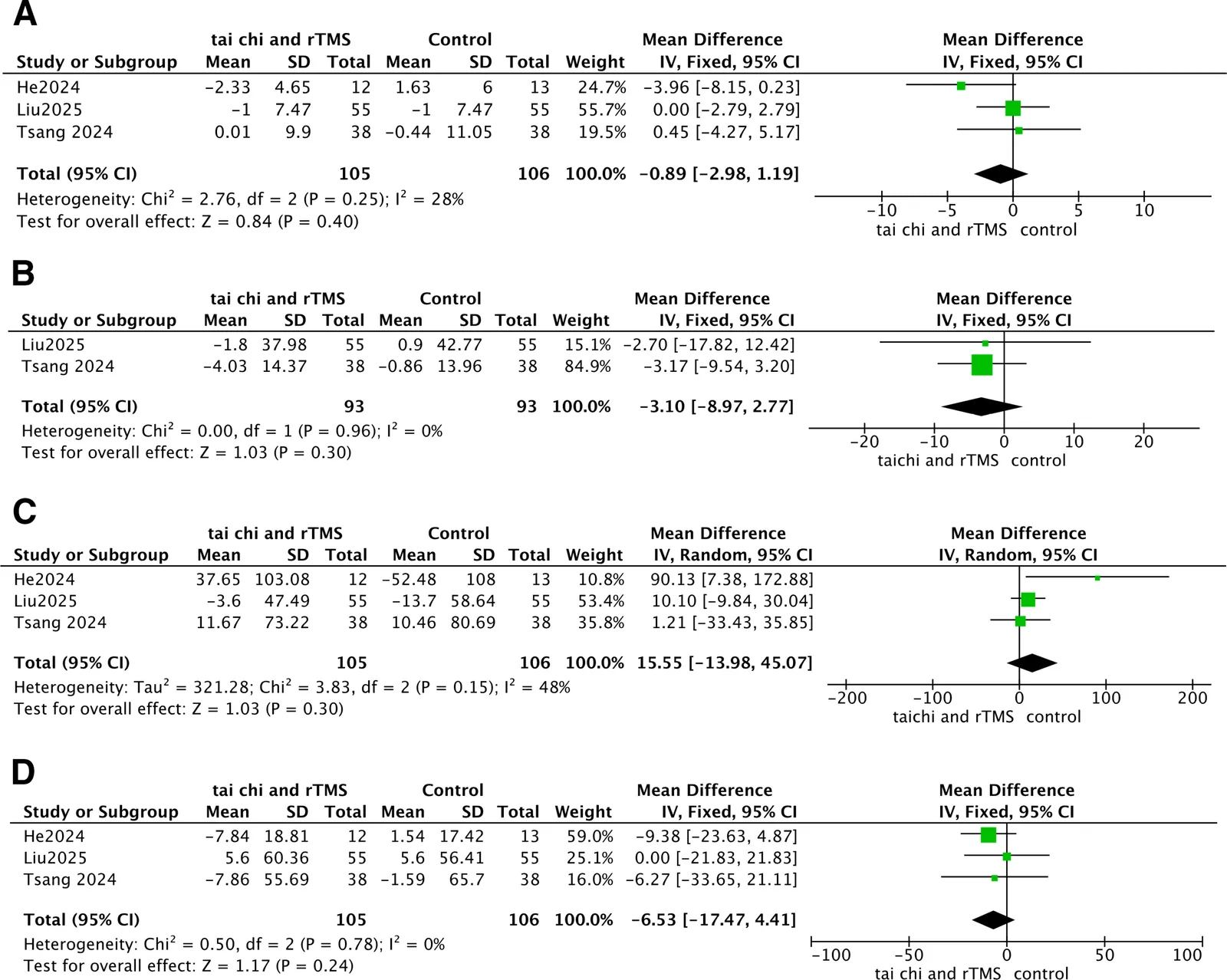
Forest plots showing the pooled effects of Tai Chi combined with rTMS on sleep-related outcomes. (A) Sleep efficiency. (B) Sleep latency. (C) Total sleep time. (D) Wake after sleep onset (WASO). rTMS = repetitive transcranial magnetic stimulation.

#### 3.5.2. Sleep latency

Two studies involving 186 participants provided usable sleep-latency data. The combined estimate was MD = −3.10 minutes (95% CI: −8.97–2.77; *P* = .30), with no observed between-study heterogeneity (*I*^2^ = 0%). Therefore, the available data did not provide clear evidence of an effect on sleep latency (Fig. 5B).

#### 3.5.3. Total sleep time

All 3 trials reported total sleep time, providing data from 211 participants. The pooled estimate was MD = 15.55 minutes (95% CI: −13.98–45.07; *P* = .30). Heterogeneity was moderate (*I*^2^ = 48%), and the confidence interval included the null value (Fig. 5C).

#### 3.5.4. Wake after sleep onset

WASO was also reported by all 3 studies. The meta-analysis produced an MD of −6.53 minutes (95% CI: −17.47–4.41; *P* = .24), with no detected heterogeneity (*I*^2^ = 0%). These results did not establish a clear difference between combined Tai Chi plus rTMS and the comparator conditions (Fig. 5D).

### 3.6. Secondary emotional outcomes

#### 3.6.1. Depression

Depression outcomes were available from all 3 studies (n = 211). The pooled standardized effect was −0.27 (95% CI: −0.75–0.21; *P* = .27), while heterogeneity was substantial (*I*^2^ = 62%). Given the confidence interval and between-study variability, the evidence regarding an effect on depressive symptoms remained inconclusive (Fig. 6A).

**Figure 6.**
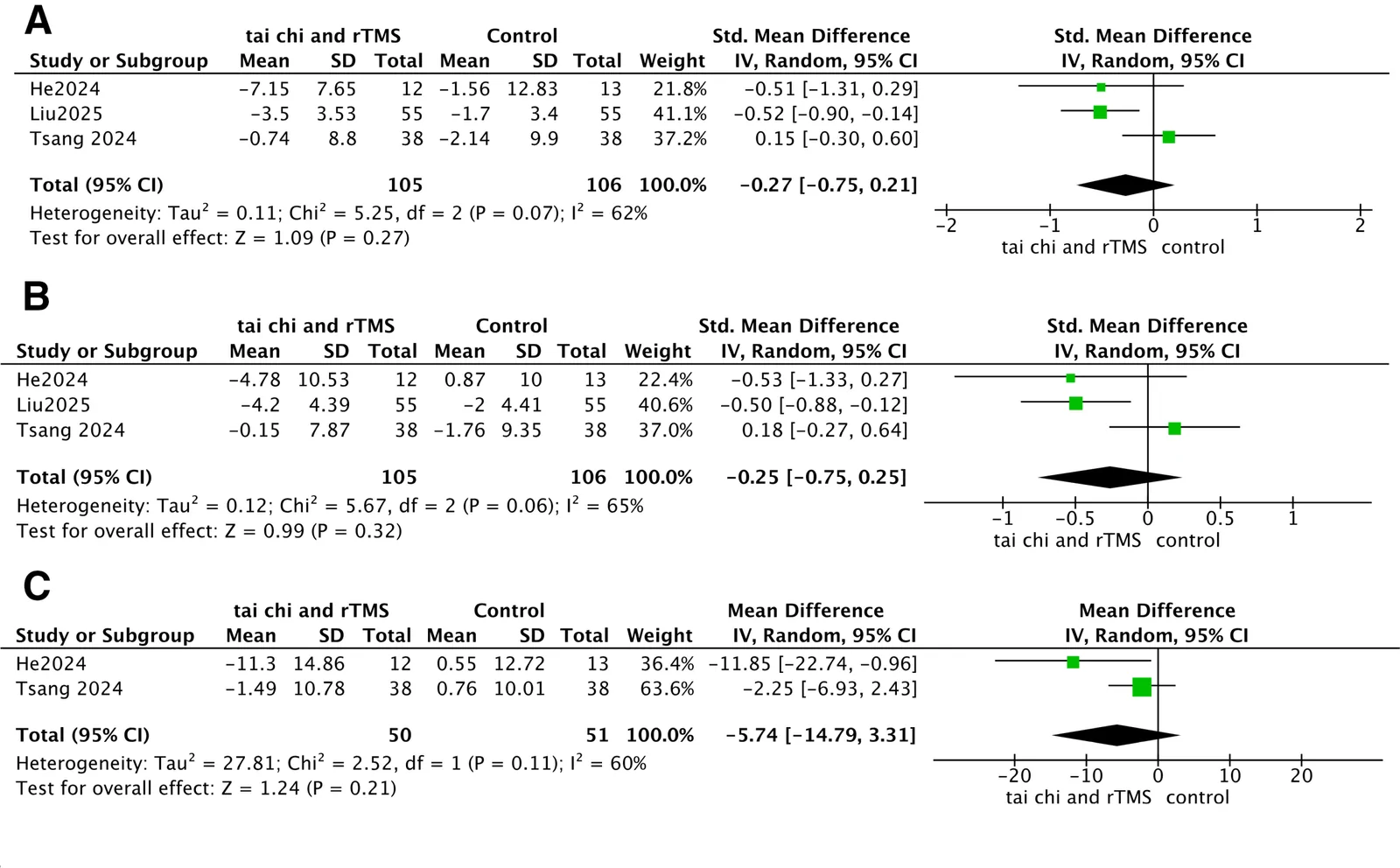
Forest plots showing the pooled effects of Tai Chi combined with rTMS on emotional outcomes. (A) Depression score. (B) Anxiety score. (C) Stress score. rTMS = repetitive transcranial magnetic stimulation.

#### 3.6.2. Anxiety

The 3 trials also contributed anxiety data for 211 participants. The combined effect estimate was SMD = −0.25 (95% CI: −0.75–0.25; *P* = .32), accompanied by substantial heterogeneity (*I*^2^ = 65%). Accordingly, the available evidence did not establish a clear effect on anxiety symptoms (Fig. 6B).

#### 3.6.3. Stress

Stress was evaluated in 2 trials comprising 101 participants. The pooled analysis yielded an MD of −5.74 (95% CI: −14.79–3.31; *P* = .21), with *I*^2^ = 60%. The wide confidence interval and between-study heterogeneity indicate considerable uncertainty regarding the effect on stress (Fig. 6C).

### 3.7. Certainty of evidence

The GRADE evaluation indicated moderate-certainty evidence for sleep severity, whereas certainty was low for the objective sleep outcomes and very low for depression, anxiety, and stress. Reductions in certainty primarily reflected imprecision, inconsistency for the emotional outcomes, and the small evidence base. No major concerns regarding indirectness were identified. The complete GRADE assessment is presented in Table 3.

**Table 3:** GRADE assessment of the certainty of evidence for included outcomes.

	Study limitation	Imprecision	Inconsistency	Indirectness	Publication bias	GRADE
Sleep severity	No downgrade	No downgrade	No downgrade	No downgrade	Downgraded because publication bias could not be reliably assessed due to the small number of studies.	Moderate
Sleep efficiency	No downgrade	Downgrade as wide 95% CI	No downgrade	No downgrade	Downgraded because publication bias could not be reliably assessed due to the small number of studies.	Low
Sleep latency	No downgrade	Downgrade as wide 95% CI	No downgrade	No downgrade	Downgraded because publication bias could not be reliably assessed due to the small number of studies.	Low
Total sleep time	No downgrade	Downgrade as wide 95% CI	No downgrade	No downgrade	Downgraded because publication bias could not be reliably assessed due to the small number of studies.	Low
WASO	No downgrade	Downgrade as wide 95% CI	No downgrade	No downgrade	Downgraded because publication bias could not be reliably assessed due to the small number of studies.	Low
Depression score	No downgrade	Downgrade as wide 95% CI	Downgrade as *I*^2^ = 62%	No downgrade	Downgraded because publication bias could not be reliably assessed due to the small number of studies.	Very low
Anxiety score	No downgrade	Downgrade as wide 95% CI	Downgrade as *I*^2^ = 65%	No downgrade	Downgraded because publication bias could not be reliably assessed due to the small number of studies.	Very low
Stress score	No downgrade	Downgrade as wide 95% CI	Downgrade as *I*^2^ = 60%	No downgrade	Downgraded because publication bias could not be reliably assessed due to the small number of studies.	Very low

CI = confidence interval, GRADE = Grading of Recommendations Assessment, Development and Evaluation, WASO = wake after sleep onset.

## 4. Discussion

This systematic review and meta-analysis synthesized randomized controlled trial evidence on the combined use of Tai Chi and rTMS for sleep disturbances in older adults. The principal finding was a significant improvement in overall sleep severity following the combined intervention. In contrast, pooled effects on individual sleep parameters, including sleep latency, total sleep time, sleep efficiency, and nighttime awakenings, did not reach statistical significance. Similarly, no significant pooled improvements were observed for depression, anxiety, or stress. Taken together, these findings suggest a potential benefit of combined Tai Chi and rTMS for overall sleep severity, whereas the evidence for individual sleep dimensions and emotional outcomes remains inconclusive.

The observed improvement in overall sleep severity is consistent with evidence from randomized trials supporting Tai Chi as a non-pharmacological intervention for late-life insomnia. In a three-arm randomized clinical trial involving 320 older adults with chronic insomnia, Siu et al found that both Tai Chi and conventional exercise improved actigraphy-assessed sleep efficiency, wake time after sleep onset, and number of awakenings compared with control conditions. Importantly, these improvements persisted at the 24-month follow-up, although objective sleep outcomes did not differ significantly between Tai Chi and conventional exercise.^[9]^ This finding is relevant to the present meta-analysis because it supports the durability and feasibility of Tai Chi while also suggesting that its effectiveness should not necessarily be interpreted as superior to conventional exercise.

Evidence comparing Tai Chi with established behavioral therapy provides additional perspective. In an earlier comparative-effectiveness randomized trial of older adults with chronic insomnia, cognitive behavioral therapy produced greater remission of clinical insomnia and more sustained improvements in several sleep-related outcomes than Tai Chi.^[10]^ Thus, although Tai Chi represents an accessible and low-impact approach, the existing literature does not support the assumption that Tai Chi alone is universally superior to other non-pharmacological treatments. Rather, its potential advantages include suitability for older adults, minimal equipment requirements, opportunities for continued self-practice, and simultaneous engagement of physical activity, breathing regulation, and attentional control. These characteristics may make Tai Chi particularly suitable as one component of a multimodal intervention.

The contribution of rTMS may help explain why the combined intervention produced a significant effect on overall sleep severity. Unlike Tai Chi, which primarily engages behavioral, physical, and psychophysiological processes, rTMS directly modifies cortical excitability and neural network activity. Recent randomized evidence supports the potential of noninvasive neuromodulation for insomnia. For example, a randomized sham-controlled trial of continuous theta-burst stimulation in patients with primary insomnia demonstrated a substantial reduction in insomnia severity and improvements in objective total sleep time and slow-wave sleep, with benefits in sleep quality persisting during follow-up.^[11]^ Although continuous theta-burst stimulation is not identical to the conventional rTMS protocols included in the present review, these findings support the broader biological plausibility of targeting cortical networks as a therapeutic strategy for sleep disturbance.

More directly relevant evidence comes from trials evaluating Tai Chi and rTMS in combination. A pilot randomized controlled trial by He et al compared Tai Chi plus rTMS with Tai Chi alone and treatment as usual in community-dwelling older adults. The authors proposed that Tai Chi and rTMS may influence different components of hyperarousal, including somatic, cognitive, and cortical arousal, thereby providing a mechanistic rationale for their combined use.^[7]^ Subsequently, a larger four-arm randomized controlled trial involving 152 participants compared Tai Chi plus active rTMS, Tai Chi plus sham rTMS, Tai Chi alone, and low-intensity physical exercise, providing more direct evidence regarding the incremental contribution of active neuromodulation when added to Tai Chi.^[6]^ These trials strengthen the rationale for considering Tai Chi and rTMS as complementary interventions rather than simply 2 interchangeable non-pharmacological treatments.

Additional evidence from older adults with sleep disorders and mild cognitive impairment further supports this possibility. In a randomized clinical trial involving 110 participants, the addition of 1-Hz rTMS to Tai Chi Chuan resulted in a greater reduction in PSQI scores than the comparator intervention and was also associated with improvement in cognitive performance. The investigators proposed that changes in neural plasticity may contribute to these effects.^[8]^ Although the population differed from that of some trials included in the present meta-analysis, these findings provide clinically relevant evidence that neuromodulation may enhance the effects of a behavioral mind–body intervention in older adults with sleep-related problems.

An important finding of the present meta-analysis was the discrepancy between the significant improvement in overall sleep severity and the nonsignificant effects on individual sleep parameters. This pattern should not necessarily be regarded as contradictory. A similar outcome-specific pattern has been observed with other multimodal non-pharmacological interventions in older populations. Although the population and intervention differ from those examined in the present review, these findings illustrate that multimodal interventions may produce measurable benefits in some domains without corresponding improvements across all related outcomes. This may provide context for our finding that combined Tai Chi and rTMS improved overall sleep severity but did not significantly affect individual sleep parameters or emotional outcomes. Global insomnia or sleep-quality instruments integrate several dimensions of the sleep experience, including perceived sleep difficulty, satisfaction, daytime consequences, and distress, whereas individual outcomes such as sleep latency or total sleep time capture narrower aspects of sleep. Moreover, subjective and objectively measured sleep improvements do not always occur in parallel. The randomized trial by Siu et al, for example, demonstrated that Tai Chi improved both subjective and actigraphy-based outcomes, but the absolute changes in objective parameters were relatively modest.^[9]^ The small number of trials in the present analysis, together with differences in intervention duration, rTMS parameters, Tai Chi protocols, and methods of sleep measurement, may therefore have limited the ability to detect effects on individual sleep components. Similar outcome-specific differences have been reported in other non-pharmacological sleep research. Cai et al found modest increases in melatonin concentrations following non-pharmacological ICU nursing interventions, whereas effects on sleep quality and cortisol remained inconclusive.^[12]^

The absence of statistically significant pooled effects on depression, anxiety, and stress also requires cautious interpretation. Emotional symptoms were not necessarily the primary clinical indication for enrollment in the included trials, and participants with relatively low baseline symptom severity may have had limited scope for improvement. Furthermore, different studies used different instruments to measure psychological outcomes. Differences between instruments such as the Hamilton rating scales and the Depression Anxiety Stress Scales are important because they assess partially different constructs, use different scoring ranges, and have different thresholds for symptom severity. This measurement heterogeneity may have contributed to the substantial between-study heterogeneity observed for depression, anxiety, and stress. Notably, neuromodulation studies have also reported differential effects across emotional outcomes; for example, the recent randomized trial of continuous theta-burst stimulation found improvement in anxiety but not depression.^[11]^ Therefore, the nonsignificant pooled psychological outcomes in our analysis should be interpreted as inconclusive rather than as evidence of no effect.

From a clinical perspective, combining Tai Chi with rTMS has both potential advantages and practical limitations. Tai Chi is inexpensive, low impact, and potentially sustainable after supervised treatment ends. The long-term randomized evidence showing maintenance of sleep benefits up to 24 months supports this potential for continued self-management.^[9]^ rTMS, however, requires specialized equipment, trained personnel, repeated clinic visits, and greater healthcare resources. Consequently, demonstrating that the combination is effective is not sufficient to establish that it should replace simpler interventions. Future trials should determine whether adding rTMS to Tai Chi produces a clinically meaningful incremental benefit relative to Tai Chi alone, conventional exercise, cognitive behavioral therapy for insomnia, or other established non-pharmacological strategies. Cost-effectiveness, adherence, treatment burden, and durability of benefit should also be evaluated.

Several limitations should be considered when interpreting the present findings. First, only a small number of randomized controlled trials were available, resulting in limited statistical power and imprecise estimates for several outcomes. Moreover, the pooled effect on overall sleep severity was sensitive to the exclusion of individual studies, further limiting the robustness of this finding. In addition, the certainty of evidence ranged from moderate for sleep severity to low or very low for the remaining outcomes, which further limits the strength of the conclusions. Second, heterogeneity existed in Tai Chi protocols, rTMS stimulation targets and parameters, treatment duration, participant characteristics, and outcome instruments. Third, depression, anxiety, and stress were assessed using different scales, which may have contributed to heterogeneity and limit direct clinical interpretation of standardized pooled effects. Fourth, the relatively small evidence base precluded reliable assessment of publication bias and meaningful subgroup or meta-regression analyses. Finally, most available trials were conducted in geographically and culturally restricted populations; therefore, the generalizability of the findings to older adults in other healthcare systems remains uncertain.

In conclusion, the available randomized evidence suggests that combined Tai Chi and rTMS may improve overall sleep severity in older adults with sleep disturbances, whereas effects on specific sleep parameters and emotional outcomes remain uncertain. The existing literature provides a plausible rationale for combining the sustained behavioral and psychophysiological effects of Tai Chi with the direct neuromodulatory effects of rTMS. However, the current evidence does not establish that the combined intervention is superior to established alternatives such as Tai Chi alone, conventional exercise, or cognitive behavioral therapy for insomnia. Larger multicenter randomized trials incorporating active comparator groups, standardized outcome measures, clearly specified stimulation protocols, longer follow-up, and economic evaluation are needed to determine the incremental clinical value of this combined approach.

## 5. Conclusion

This systematic review and meta-analysis suggest that Tai Chi combined with rTMS may improve overall sleep severity in older adults with sleep disturbances. However, evidence for benefits on objective sleep parameters and emotional outcomes remains limited and inconclusive. Given the small number of available trials, modest sample sizes, and heterogeneity in outcome assessment, these findings should be interpreted with caution. Tai Chi may be considered a safe foundational intervention, while rTMS could serve as an adjunctive option for individuals with persistent symptoms under specialist supervision. Future large-scale, well-designed randomized controlled trials with standardized outcome measures and longer follow-up are needed to clarify the clinical role of this combined intervention.

## Author contributions

**Conceptualization:** Mengxia Xu.

**Data curation:** Mengxia Xu.

**Formal analysis:** Mengxia Xu, Jianxia Zhuang.

**Investigation:** Mengxia Xu, Jianxia Zhuang.

**Methodology:** Mengxia Xu, Shengen Zheng.

**Project administration:** Shengen Zheng.

**Resources:** Shengen Zheng.

**Software:** Shengen Zheng.

**Supervision:** Shengen Zheng.

**Validation:** Shengen Zheng.

**Visualization:** Shengen Zheng.

**Writing – original draft:** Mengxia Xu, Yu Wang, Zhoujian Ding, Jianxia Zhuang, Shengen Zheng.

**Writing – review & editing:** Mengxia Xu, Yu Wang, Zhoujian Ding, Jianxia Zhuang, Shengen Zheng.



## References

[1] Wang J Yuan Y Bai Y. Non-pharmacological interventions for improving sleep quality in adults aged 50 years and older: a systematic review and network meta-analysis. Arch Public Health. 2026;84. 10.1186/s13690-026-01892-5 PMC13188796 41981687

[2] Lyu L Li Y Fan X Seo J Eunna O. The effect of Tai Chi exercise intervention on sleep quality in community residing older adults: a systematic review and meta-analysis. Eur Geriatr Med. 2024;15:381–96. 10.1007/s41999-023-00923-6 38227110

[3] Khosravi M Khosravi R. Effectiveness of non-invasive brain stimulation in improving sleep quality in insomnia: a systematic review. Sleep Science and Practice. 2025;9:18.

[4] Zheng B Cai P Chen Y Wu Y Liu H. PADI4 and IL-33 gene polymorphisms with susceptibility to systemic lupus erythematosus and juvenile idiopathic arthritis, a systematic review and meta-analysis. Medicine (Baltim). 2023;102:e33700. 10.1097/MD.0000000000033700 PMC10158862 37145011

[5] Higgins JPT TJ Chandler J Cumpston M Li T Page MJ.. Cochrane handbook for systematic reviews of interventions. 2nd ed. New York: Wiley;. 2019.

[6] He J Chan SHW Chung RCK Tsang HWH. Effect of combined Tai Chi and repetitive transcranial magnetic stimulation for sleep disturbance in older adults: a randomized controlled trial. J Psychiatr Res. 2024;180:281–90. 10.1016/j.jpsychires.2024.10.025 39476538

[7] He J Chan SHW Lin J Tsang HWH. Integration of Tai Chi and repetitive transcranial magnetic stimulation for sleep disturbances in older adults: a pilot randomized controlled trial. Sleep Med. 2024;122:35–44. 10.1016/j.sleep.2024.07.029 39121822

[8] Liu Z Zhang L Bai L. Repetitive transcranial magnetic stimulation and Tai Chi Chuan for older adults with sleep disorders and mild cognitive impairment: a randomized clinical trial. JAMA Network Open. 2025;8:e2454307. 10.1001/jamanetworkopen.2024.54307 PMC12548080 39792383

[9] Siu PM Yu AP Tam BT. Effects of Tai Chi or exercise on sleep in older adults with insomnia: a randomized clinical trial. JAMA Network Open. 2021;4:e2037199–e99. 10.1001/jamanetworkopen.2020.37199 PMC7885034 33587135

[10] Irwin MR Olmstead R Carrillo C. Cognitive behavioral therapy vs. Tai Chi for late life insomnia and inflammatory risk: a randomized controlled comparative efficacy trial. Sleep. 2014;37:1543–52. 10.5665/sleep.4008 PMC4153053 25142571

[11] Zhu X Tabarak S Que J. Efficiency and safety of continuous theta burst stimulation for primary insomnia: a randomized clinical trial. Sleep Med. 2024;124:77–83. 10.1016/j.sleep.2024.09.006 39276701

[12] Cai D Zheng X Zheng B Ye H. Non-pharmacological ICU nursing interventions and melatonin concentrations: a systematic review and meta-analysis of randomized controlled trials. BMC Nurs. 2026;25. 10.1186/s12912-026-04938-9 PMC13551963 42343364

