# Supplementary material for: Effects of Tai Chi combined with repetitive transcranial magnetic stimulation on sleep and emotional outcomes in older adults: A systematic review and meta-analysis of randomized controlled trials
Source: Medicine (Baltimore). 2026 Nov 6;105(45):e51253. doi: 10.1097/MD.0000000000051253 (PMC13747490; doi:10.1097/MD.0000000000051253)
Supplement: Supplementary file 1 [file medi-105-e51253-s001.docx]

Supplementary Appendix 1. Search strategies for all databases (from inception to 12.16.2025)

| Database | Search Strategy |
| --- | --- |
| Pubmed | (((((ageing[MeSH Terms]) OR (older[Title/Abstract])) OR (elder[Title/Abstract])) AND (randomized[Title/Abstract])) AND (tai chi[Title/Abstract])) AND (repetitive transcranial magnetic stimulation[Title/Abstract]) |
| Scopus | TITLE-ABS-KEY(ageing OR older OR elder)  AND TITLE-ABS-KEY(randomized)  AND TITLE-ABS-KEY("tai chi")  AND TITLE-ABS-KEY("repetitive transcranial magnetic stimulation") |
| Embase | ('aging'/exp OR aging:ti,ab OR ageing:ti,ab OR aged:ti,ab  OR older:ti,ab OR elder*:ti,ab OR elderly:ti,ab  OR 'older adult*':ti,ab OR 'older people':ti,ab)  AND  (random*:ti,ab OR trial*:ti,ab OR placebo*:ti,ab  OR 'controlled trial':ti,ab OR 'clinical trial':ti,ab)  AND  ('tai chi'/exp OR 'tai chi':ti,ab OR taichi:ti,ab  OR 'tai ji':ti,ab OR taiji:ti,ab  OR 'tai chi chuan':ti,ab OR 'tai ji quan':ti,ab)  AND  ('transcranial magnetic stimulation'/exp  OR 'repetitive transcranial magnetic stimulation':ti,ab  OR 'transcranial magnetic stimulation':ti,ab  OR rTMS:ti,ab  OR TMS:ti,ab) |
| Cochrane | #1 (ageing OR older OR elder):ti,ab,kw  #2 randomized:ti,ab,kw  #3 "tai chi":ti,ab,kw  #4 "repetitive transcranial magnetic stimulation":ti,ab,kw  #5 #1 AND #2 AND #3 AND #4 |
| Web of Science | (TI=(ageing OR older OR elder) OR AB=(ageing OR older OR elder))  AND (TI=(randomized) OR AB=(randomized))  AND (TI=("tai chi") OR AB=("tai chi"))  AND (TI=("repetitive transcranial magnetic stimulation") OR AB=("repetitive transcranial magnetic stimulation")) |
| CINAHL | (MH "Aging+" OR TI (ageing OR older OR elder) OR AB (ageing OR older OR elder))  AND  (TI randomized OR AB randomized)  AND  (TI "tai chi" OR AB "tai chi")  AND  (TI "repetitive transcranial magnetic stimulation" OR AB "repetitive transcranial magnetic stimulation") |
